# A novel alkane monooxygenase evolved from a broken piece of ribonucleotide reductase in *Geobacillus kaustophilus* HTA426 isolated from Mariana Trench

**DOI:** 10.1007/s00792-024-01332-8

**Published:** 2024-02-14

**Authors:** Tanasap Nithimethachoke, Chanita Boonmak, Masaaki Morikawa

**Affiliations:** 1 Graduate School of Environmental Science, Hokkaido University, Kita-10 Nishi-5, Kita-ku, Sapporo, 060-0810 Japan; 2 Department of Microbiology, Faculty of Science, Kasetsart University, 50 Ngam Wong Wan Rd., Lat Yao, Chatuchak, Bangkok, 10900 Thailand

**Keywords:** Alkane degradation, Alkane monooxygenase, *Geobacillus kaustophilus*, Ribonucleotide reductase, R2lox protein

## Abstract

We have accidentally found that a thermophilic *Geobacillus kaustophilus* HTA426 is capable of degrading alkanes although it has no alkane oxygenating enzyme genes. Our experimental results revealed that a putative ribonucleotide reductase small subunit *Gk*R2loxI (GK2771) gene encodes a novel heterodinuclear Mn–Fe alkane monooxygenase/hydroxylase. *Gk*R2loxI protein can perform two-electron oxidations similar to homonuclear diiron bacterial multicomponent soluble methane monooxygenases. This finding not only answers a long-standing question about the substrate of the R2lox protein clade, but also expands our understanding of the vast diversity and new evolutionary lineage of the bacterial alkane monooxygenase/hydroxylase family.

## Introduction

The ability of microorganisms to degrade chemically stable alkanes and their use as carbon sources have been of interest for more than a century. Alkane-degrading bacteria are found in various environments, such as terrestrial, subsurface, or hydrosphere environments, in areas that are constantly or previously exposed to petroleum hydrocarbons (Van Beilen et al. 2003; Wentzel et al. 2007). *Geobacillus* genus includes low GC Gram-positive, rod-shaped, spore-forming bacteria that have 55–65 °C optimum growth temperatures. Due to its easy cultivation and high growth rate, *Geobacillus* species are used to industrially produce thermostable enzymes that cannot be obtained from mesophilic organisms (Hussein et al. 2015; Kananavičiūtė and Čitavičius 2015). Several *Geobacillus* species utilize alkanes and other hydrocarbons (Meintanis et al. 2006; Elumalai et al. 2017). These include multiple strains of *Geobacillus stearothermophilus* (Sokhoh et al, 1993; Sun et al. 2015; Zhou et al. 2018), *Geobacillus jurassicus* DS1 (Nazina et al. 2005), *Geobacillus kaustophilus* TERI NSM (Sood and Lal 2008), *Geobacillus thermodenitrificans* NG80-2 (Feng et al. 2007), and *Geobacillus thermoleovorans* B23 (Kato et al. 2001; Boonmak et al. 2014) that degrade medium- to long-chain alkanes. The alkane degradation reaction starts with hydroxylation by a monooxygenase/hydroxylase, followed by a two-step oxidation process by alcohol dehydrogenase and aldehyde dehydrogenase. Alkane monooxygenases are generally classified into heme iron-dependent P450 type oxidoreductase CYP153 group, non-heme iron-dependent oxygenase AlkB group, and FMN-dependent non-metal oxygenase LadA group. The alkane monooxygenase/hydroxylase of *Geobacillus* is non-heme iron-dependent AlkB-type or FMN-dependent LadA-type (Wang and Shao 2013; Nie et al. 2014), while no heme iron-dependent CYP153 type alkane monooxygenase has not been reported yet (Padayachee et al. 2020).

*Geobacillus kaustophilus* HTA426 was isolated from the deepest sea sediment of the Mariana Trench, at a depth of 10, 897 m (Takami et al. 2004). Our previous study suggested that *G. kaustophilus* MK9, a *pyrF* deletion HTA426 mutant, can degrade alkanes (Boonmak 2014); however, we could not find AlkB- or LadA-encoding genes in HTA426 genome (GenBank: BA000043.1) to support this claim. *pyrF* is a gene encoding orotidine 5′-monophosphate decarboxylase that does not relate to alkane degradation metabolisms. Here, we found that a gene, *gk2771*, whose product annotated as a putative ribonucleotide reductase small subunit RNR2 (GK2771), is located at a locus far from the complete ribonucleotide reductase gene set *gk1911* and *gk0912* encoding large and small subunits RNR1/RNR2, respectively. This maverick gene *gk2771* forms a cluster with the genes encoding aldehyde dehydrogenase (*gk2772*, GK2772) and alcohol dehydrogenase (*gk2774*, GK2774), which are essential for alkane metabolism (Ji et al. 2013). GK2771 location strongly implies its involvement in alkane metabolism rather than in deoxyribonucleotide synthesis.

Ribonucleotide reductase has been extensively studied because it is an essential enzyme for de novo deoxyribonucleotide synthesis in all living organisms (Andrews 2010; Munro et al. 2013). Class I RNRs are typically characterized by their oxygen requirements for forming stable tyrosyl radicals using a catalytic metal center. In contrast, class II RNRs are indifferent to oxygen and form a thiyl radical using S-adenosylcobalamin. Class III RNRs are anaerobic and form a glycyl radical using 4Fe-4S center and S-adenosylmethionine (Nordlund and Reichard 2006). Class I RNRs are further subdivided into classes Ia–Ie. Class Ia enzymes utilize a characteristic diiron, Fe–Fe, metal cofactor, which reacts with oxygen to generate stable tyrosyl radicals. In contrast, class Ib enzymes utilize a dimanganese, Mn–Mn metal cofactor with a tyrosyl radical, and class Ic enzymes, which lack the tyrosyl radical and diiron site, utilize a heterodinuclear Mn–Fe metal cofactor (Cotruvo and Stubbe 2011). Subclasses Id and Ie have also been discovered recently, while class Id also utilizes dimanganese Mn–Mn center, but no tyrosyl radical, is involved (Rose et al. 2018). The peculiar class Ie lacks a metal cofactor and uses a tyrosine-derived dihydroxyphenylalanine (DOPA) radical (Srinivas et al. 2018; Blaesi et al. 2018). Class I RNRs generally consist of two different subunits that form the RNR1_n_RNR2_2_ structure, where the number of subunits (n) can be two or six (Rofougaran et al. 2006). It is noteworthy that the small subunit, RNR2, and soluble methane monooxygenase (sMMO) that oxidizes methane to methanol have been referred as “twin enzyme” due to their significant similarities in the metal coordinating active site structure (Que and Dong 1996; Torrent et al. 2002). Several genes in the archaeal and bacterial genomes were misannotated as RNR2-encoding genes because of the translated amino acid sequences are significantly similar. Sequence similar proteins but least probable RNR2 are referred to as “R2-like ligand binding oxidases” (R2lox) (Andersson and Högbom 2009). GK2771 of *G. kaustophilus* HTA426 is also known as *Gk*R2loxI. The crystal structure of *Gk*R2loxI, 4HR0_A, has already been reported, and is the first R2lox homolog in the genus *Geobacillus* (Griese et al. 2013). Interestingly, the recombinant *Gk*R2loxI produced in *E. coli* was co-purified with C16 or C18 hydroxy fatty acids (Griese et al. 2013; Diamanti et al. 2022). Based on this information, we hypothesized that *Gk*R2loxI (GK2771) functions as a novel alkane hydroxylase in the alkane degradation system.

In this study, the alkane degradation activity of *G. kaustophilus* HTA426 was first confirmed. Second, the open reading frame of GK2771 was PCR-amplified and cloned into the pET-28a vector for heterologous protein production in *E. coli*, followed by detection of alkane degradation activity using the crude GK2771 enzyme and recombinant *E. coli* cell lysate. We discovered a novel alkane monooxygenase/hydroxylase that may have evolved specifically in high-temperature environments.

## Materials and methods

### Bacterial strains and plasmids

*G. kaustophilus* HTA426 (JCM 12893) was grown in nutrient L-broth, LB, or basal mineral medium, LBM (Kato et al. 2001) at 60 °C with or without shaking at 120 rpm for alkane degradation assay. *Escherichia coli* DH5α and *E. coli* BL21 (DE3) were used as host cells for gene cloning and recombinant protein production and grown in LB at 37 °C with shaking at 120 rpm. pUC19 containing the ampicillin resistance gene was used as the cloning vector plasmid. pET-28a, which harbored a kanamycin resistance gene, was used as the gene overexpression plasmid. *E. coli* carrying the plasmid was cultured in LB supplemented with 100 µg/mL ampicillin or 50 µg/mL kanamycin.

### GK2771 gene cloning

The genomic DNA of *G. kaustophilus* HTA426 was prepared using the InstaGene^™^ Matrix (Bio-Rad Laboratories, Inc.). First, the open reading frame (ORF) of *gk2771* containing *the Nco*I and *Eco*RI sites was PCR-amplified using KOD Plus Neo (Toyobo Co., Ltd.) and cloned into the *Sma*I site of pUC19. However, the subcloning of the *Nco*I–*Eco*RI fragment into pET-28a was unsuccessful even after multiple trials. The *gk2771* was then directly amplified using a new forward primer aimed at blunt-end ligation to pET-28a, although an additional ATG codon was introduced at the N–terminus of GK2771. The PCR product (blunt end) was purified using a QIAquick PCR Purification Kit (Qiagen). *Nco*I- and *Eco*RI-digested pET-28a (sticky end) was ligated to the blunt end using a DNA blunting kit (TAKARA BIO Inc.). The product pET-28a–*gk2771* was transferred to *E. coli* DH5α. The correct direction of *gk2771*, with no PCR errors, was confirmed by sequencing and was subsequently transferred pET-28a–*gk2771* to *E. coli* BL21 (DE3).

### Introduction of double mutations to GK2771

The Glu202 and His205 residues, which are responsible for binding Fe atoms at the active site of GK2771 (Griese et al. 2013), were mutated. The codon sequences of the active site residues glutamic acid (GAA, E202) and histidine (CAC, H205) of *GK2771* were simultaneously changed to two alanine residues (GCA, GCC; E202A/H205A) in pET-28a-*gk2771* using the KOD Plus Mutagenesis Kit (Toyobo Co., Ltd.). The mutated pET-28a-*gk2771* plasmid was transferred to *E. coli* DH5α and the direction of insertion with no PCR error was verified by sequencing. Subsequently, the *E. coli* BL21 (DE3) cells were transformed.

### Primer sets used for PCR

F(GK2771) 5′-ATGGTCCATCATGACGG-3′ for *Nco*I blunt-end ligation at initiating codon, R(GK2771) 5′-CGGAATTCTCATGACTCAGCAGCC-3′ for *Eco*RI site after stop codon, F(A202/A205) 5′-CTGAATGGCCCGCCCTGCGTCCATGTTC-3′, and R(A202/A205) 5′-CATGGACGCAGGGCGGGCCATTCAGTTCGG-3′ were used for mutation of iron ligand amino acid residues E202 and H205.

### Recombinant GK2771 and mutant GK2771 (E202A/H205A) protein overproduction

An overnight recombinant *E. coli* BL21 (DE3) pre-culture was prepared in LB supplemented with kanamycin by shaking at 120 rpm and 37 °C. The main culture was prepared by inoculating a 1% (v/v) pre-culture and further shaking until it reached mid-log phase (OD_600_: 0.5 after 2–3 h), treated with 0.1 mM IPTG for 4 h to induce gene expression, and then harvested. The *E. coli* culture was centrifuged at 13,000×*g* and 4 °C for 10 min and the cells were washed with 20 mM Tris–HCl buffer (pH 8.0) twice. The cell pellet was resuspended in the same buffer and sonicated on ice using a Branson Ultrasonics™ sonifier S-250A (Emerson Electric Co.) for 2 min with 30 s on–off intervals. Then, the sonicated sample was centrifuged at 15,000×*g* and 4°C for 10 min to separate the cell debris and supernatant. The cell debris resuspended in Tris–HCl buffer and supernatant were used as the insoluble and soluble fractions, respectively. The protein concentration of each fraction was measured using the Bradford Protein Assay kit (Bio-Rad), and the GK2771 and mutant GK2771 (E202A/H205A) protein bands were confirmed using 15% SDS–PAGE.

### Alkane degradation test

#### Using *G. kaustophilus* HTA426 cells:

The cells were precultured overnight in LB by shaking at 60 °C and 120 rpm. The main culture was prepared by inoculating a 1% (v/v) pre-culture with overnight shaking under the same conditions. Cells were harvested by centrifugation at 13,000×*g* and 4 °C for 10 min, washed twice by fresh LB or LBM, and resuspended in an appropriate volume of LB or LBM to adjust the OD_600_ to 5.00. Then, a cell suspension aliquot in 10 mL LB or 1.5 mL LBM was transferred to sterile 100 mL or 20 mL glass vials (Maruemu Corporation) with 0.1% (v/v) standard oil (Tokyo Chemical Industry Ltd.), respectively. The glass vials were sealed with a butyl rubber stopper and an aluminum crimp, shaken once daily to mix the contents, and incubated at 60 °C without shaking. All experiments were performed in triplicate.

#### Using recombinant GK2771 and mutant GK2771 (E202A/H205A) proteins:

After the recombinant proteins were obtained as crude enzymes in a soluble form, the total protein concentration was measured using a Bradford protein assay kit (Bio-Rad Laboratories, Inc.). A 1.5 mL reaction mixture was prepared in 20 mL glass vial containing 20 mM Tris–HCl buffer (pH 8.0), 10 mg/mL crude enzyme, 0.1 mM NADH, 0.1 mM each of MnSO_4_/FeSO_4_, and 1.5 µL (0.1% v/v) standard gas oil (S0526, Tokyo Chemical Industry Co. Ltd.). The vials were sealed with a butyl rubber stopper and an aluminum crimp, vigorously shaken, and incubated at 60 or 37 °C for 24 h without shaking. All experiments were performed in triplicate.

After incubating the vials for the designated periods, the remaining alkanes were extracted with 1:1 hexane:acetone containing 40 ppm biphenyl as an internal standard. The vials were vigorously shaken using a strong shaker SR-2 DS (TAITEC Corporation) at 300 strokes/min for 30 min. After resting the vials at room temperature for 1 h, 1 µL solvent was used for gas chromatography (GC/FID). The percentage of each alkane (%) in the sample was calculated from the peak area of each alkane relative to that of biphenyl.

$${\text{Remaining alkane }}\left( {\text{\%}} \right) = \frac{{\text{relative peak area of alkane in the sample}}}{{{\text{relative peak area of alkane at }}0{\text{ h of parallely prepared sample}}}}$$

### Gas chromatography

The GC/FID (GC-2014, SHIMADZU Corporation) system was equipped with a flame ionization detector and a HP-5 (Agilent Technologies, Inc., USA) non-polar capillary column (30 m length, 0.32 mm diameter, 0.25 µm film thickness). Helium was used as a carrier gas at 25 mL/min flow rate. The temperature increased from 50 °C to 300 °C within 25 min and held at 300 °C for 10 min.

## Results and discussion

### Alkane degradation test using G. kaustophilus HTA426

**Fig. 1:**
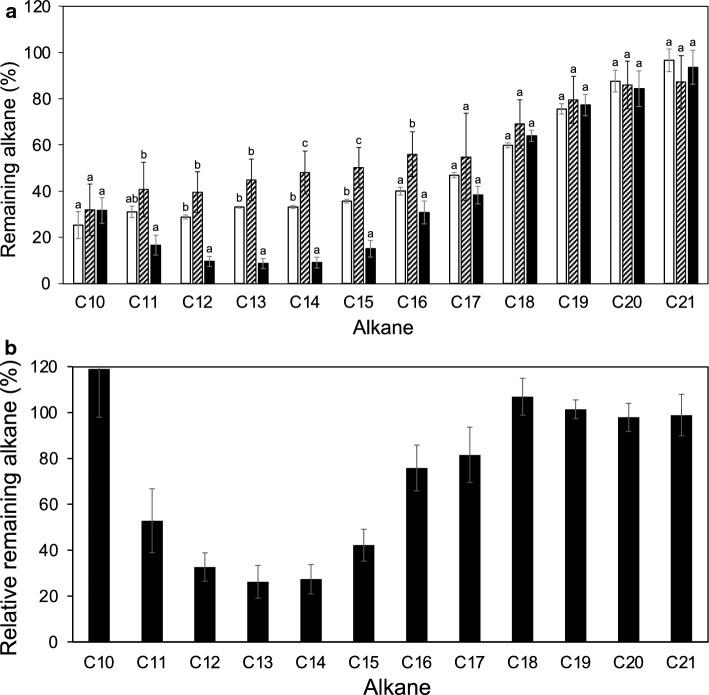
Alkane degradation by HTA426 in nutrient broth, LB culture condition at 60 °C on day 5. **a** Comparison of remaining alkane amount in vials containing no-cell control, autoclaved HTA426 cell control, and HTA426 cells on day 5 at 60 °C relative to that in the 0 d sample. Open bar, no-cell negative control; hatched bar, autoclaved HTA426 cell negative control; closed bar, HTA426 cells. **b** Relative remaining alkane amounts in vials containing HTA426 culture and no-cell blank control. Significance test was performed using one-way ANOVA followed by Tukey’s post hoc test by SPSS. The letters indicate statistically significant (*P* < 0.05) differences

In nutrient broth culture condition:

*Geobacillus kaustophilus* HTA426 clearly degraded alkanes with carbon numbers ranging from C11 to C18 in nutrient broth, LB (Fig. 1). Degradation was detectable after 1 day till 5 day. The remaining alkane amounts in the vials containing HTA426 cells were reduced to 53% and 41% (C11), 33% and 24% (C12), 26% and 19% (C13), 27% and 19% (C14), 42% and 30% (C15), 76% and 55% (C16), 82% and 70% (C17), and 107% and 93% (C18) compared to those in the vials containing no-cell control and autoclaved HTA426 cells, respectively, after incubating for 5 days at 60 °C. The amount of alkanes, particularly short-chain alkanes, such as C10 (25%) and C12 (28%), also naturally reduced in the no-cell control vials (Fig. 1a). This is probably due to alkane evaporation and adsorption on the butyl rubber stopper in the vials. The reduction in alkanes in the bottle containing autoclaved HTA426 cells was even lesser than that in the bottle containing no-cell control. This may be because dead cells bind to and trap alkanes, reducing their evaporation loss. Replacing the headspace air in the vials with 100% oxygen did not increase the alkane degradation activity of HTA426 (data not shown). These results suggest that atmospheric oxygen was sufficient for alkane degradation under these culture conditions.

**Fig. 2:**
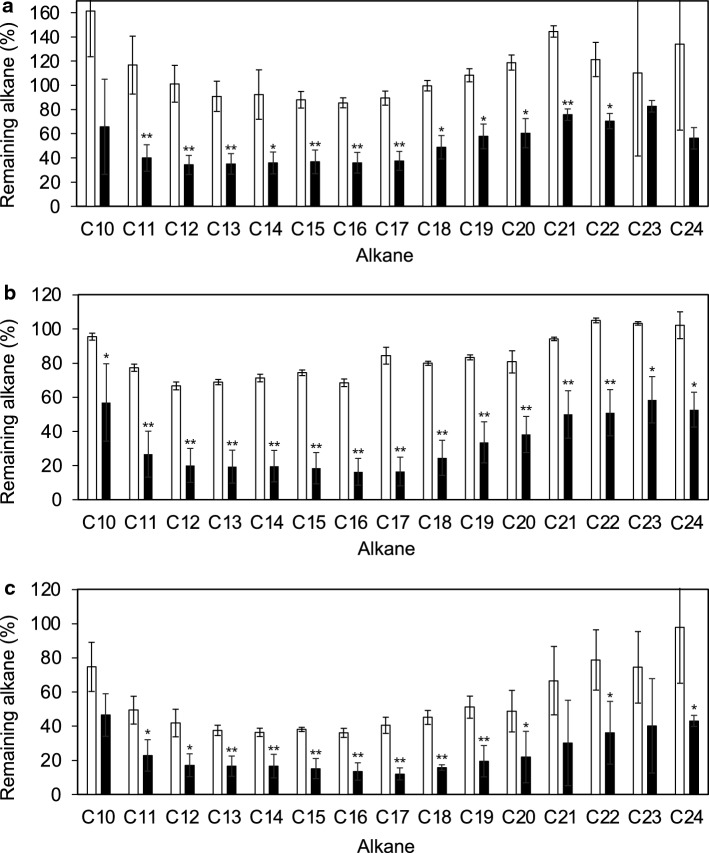
Alkane degradation by HTA426 in mineral medium, LBM culture condition at 60 °C. Comparison of the remaining amounts of alkanes between vials containing HTA426 cells and the no-cell control on days (**a**) 1, (**b**) 3, and (**c**) 7. Single asterisk: *P* < 0.05, double asterisk: *P* < 0.01

In basal mineral medium culture condition:

The alkane degradation activity of *G. kaustophilus* HTA426 was also tested in basal mineral medium under LBM conditions using a mixture of alkanes as the sole carbon source (Fig. 2). HTA426 degraded various alkanes, from C10 to C24 (Fig. 1). The reduction in the amount of alkanes was more significant for C18–C24 alkanes. Notably, a minor C9 alkane was not degraded even under these conditions. This is probably due to its low binding affinity for the alkane monooxygenase/hydroxylate enzyme (data not shown).

### Possibility that GK2771 functions as an alkane monooxygenase

As mentioned in Introduction, GK2771 belongs to the R2lox protein family, namely, *Gk*R2loxI. Owing to the significant sequence similarity of R2lox proteins, they are often misannotated as RNR2 proteins. In fact, the HTA426 genome has a complete set of ribonucleotide reductase genes, *gk0911* and *gk0912* (GCA_000009785.1), which encode the functional large subunit RNR1, GK0911 and small subunit RNR2, GK0912, respectively. Substrates for R2lox proteins have not been determined yet; however, the R2lox protein in *Mycobacterium tuberculosis* is possibly an ancestor of RNR2s and multicomponent monooxygenases (Andersson and Högbom 2009). Moreover, a R2lox-encoding gene is upregulated in the hydrocarbon-degrading haloarchaeon *Halorientalis hydrocarbonoclasticus* IM1011 when cultured in the presence of hexadecane and C16 alkanes, suggesting a role for R2lox in alkane biodegradation (Kumar et al. 2020).

**Fig. 3:**
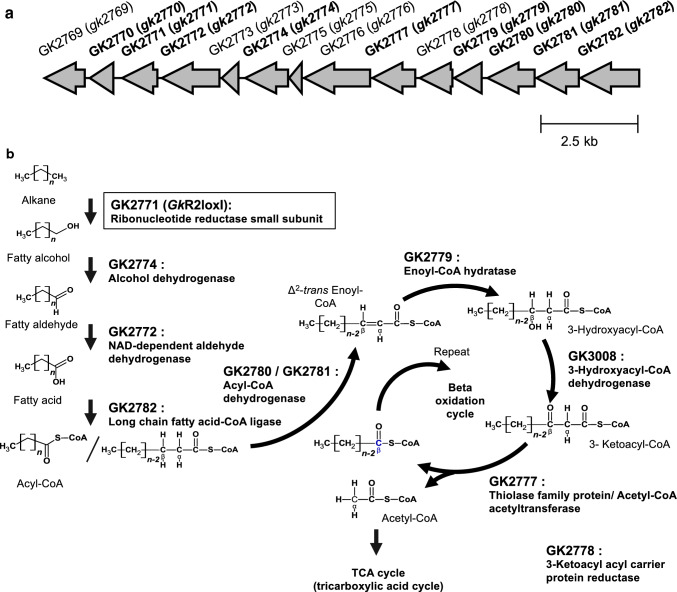
**a** A gene cluster GK2769 (*gk2769*)–GK2782 (*gk2782*) in the HTA426 genome. **b** Estimated function of each gene product

When the *G. kaustophilus* HTA426 genome, GCA_000009785.1, was carefully re-examined, we found that *gk2771* and its flanking region forms a large gene cluster encoding proteins responsible for alkane metabolism including beta-oxidation (Fig. 3). These are GK2771, putative alkane monooxygenase; GK2774, alcohol dehydrogenase; GK2772, aldehyde dehydrogenase; GK2782, long-chain fatty acid CoA ligase; GK2780/GK2781 (GK3006), GK2779, enoyl-CoA hydratase; acyl-CoA dehydrogenase; GK2778, 3-oxoacyl carrier protein reductase; enoyl-CoA hydratase; (GK3008), 3-hydroxyacyl-CoA dehydrogenase; and GK2777 (GK3007), thiolase/acetyl-CoA acetyltransferase. Surprisingly, the hypothetical proteins GK2770 and GK3040 were orthologous to P21 (99.5% identity, 200/201 aa) and P16 (94.4% identity, 185/196 aa), which are alkane-inducible membrane transporters in *G. thermoleovorans* B23 (Kato et al. 2009). Recently, P21 (UniProtKB, A0A098L0L6) and P16 (UniProtKB, A0A098L147) tertiary structures were modeled (Jumper et al. 2021), which share a similar large beta-barrel with the N-terminal alpha–helix extension structures (https://www.uniprot.org/). Considering above information, we hypothesized that GK2771 (*Gk*R2loxI) is a missing alkane monooxygenase/hydroxylase in *G. kaustophilus* HTA426.

### Gene cloning, expression and production of recombinant GK2771

**Fig. 4:**
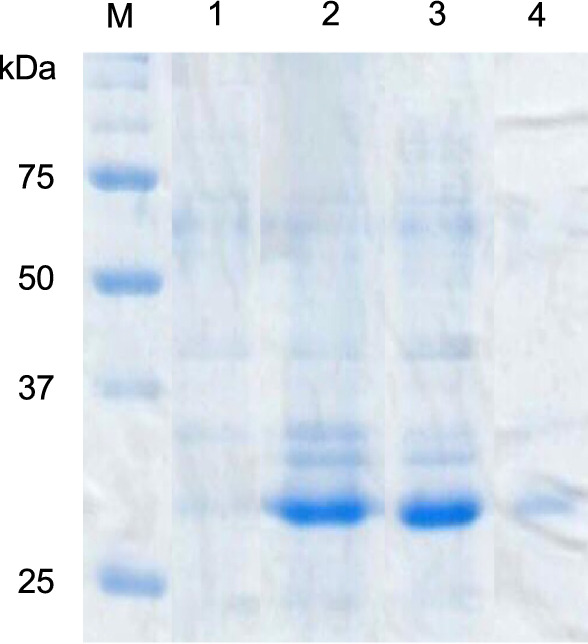
SDS–PAGE analysis of recombinant *E. coli* BL21(DE3)-produced proteins**.** Lane M, size marker; Lane 1, pET-28a vector-only total protein; lane 2, pET-28a–*gk2771* plasmid total protein; lane 3, pET-28a–*gk2771* plasmid soluble fraction; lane 4, pET-28a–*gk2771* plasmid insoluble fraction. A 30 kDa protein is uniquely produced by *gk2771* expressing *E. coli* mostly in a soluble fraction

A GK2771 ORF was PCR-amplified and cloned between *Nco*I and *Eco*RI sites of the gene expression vector pET-28a. *E. coli* BL21 (DE3) cells harboring pET-28a-*GK2771* successfully produced the recombinant protein in a soluble form upon gene induction with 0.1 mM IPTG. SDS–PAGE showed that the recombinant GK2771 protein migrated at a position estimated at 30 kDa, which is smaller than that calculated from the protein sequence, 35.2 kDa with pI 5.59 (Fig. 4).

### Alkane degradation test using crude recombinant GK2771 and mutant GK2771 (E202A/H205A) enzymes

**Fig. 5:**
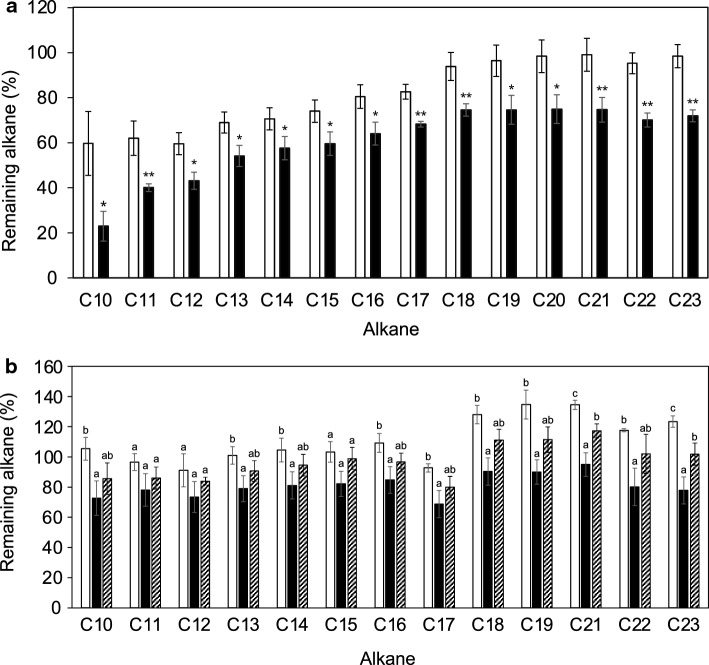
Alkane degradation by GK2771 and mutant GK2771 (E202A/H205A) enzymes denoted by remaining alkane amounts. **a** Alkane degradation activity of GK2771 at 37 °C for 24 h. Autoclaved cell lysates were used as negative controls. Open bar, autoclaved GK2771-producing *E. coli* cell lysate; Closed bar, GK2771-producing *E. coli* cell lysate. Single asterisk: *P* < 0.05, Double asterisk: *P* < 0.01 **b** Alkane degradation activity of GK2771 and mutant GK2771 (E202A/H205A) at 37 °C for 24h. The cell lysate of *E. coli* containing only the vector was used as a negative control. Open bar, cell lysate of *E. coli* containing vector only; closed bar, GK2771-producing *E. coli* cell lysate; Hatched bar, mutant GK2771 (E202A/H205A)-producing *E. coli* cell lysate. Significance test was performed using one-way ANOVA followed by Tukey’s post hoc test by SPSS. The letters indicate statistically significant (*P* < 0.05) differences

Alkane degradation by crude recombinant GK2771 was examined at 60 and 37 °C. It was found that the activity was not significantly different at these reaction temperatures. This can be attributed to the thermal instability of the host cell-originating cofactors necessary for alkane hydroxylation, such as oxidoreductases for NADH, thioredoxin, or ferredoxin. The remaining alkane amounts after treatment with crude GK2771 for 24 h were reduced to 38% (C10), 72% (C12), 81% (C14), 80% (C16),80% (C18), 76% (C20), 74% (C22) in comparison to that after treatment with autoclaved cell lysate for 24 h at 37 °C (Fig. 5a). We also observed that cell lysates of *E. coli* harboring the vector plasmid alone did not reduce the alkanes notably after the reaction (data not shown).

To confirm that the heterodinuclear Mn–Fe catalytic center is responsible for the alkane degradation reaction, two metal ligand amino acid residues, E202 and H205, out of eight (E69, V72, E102, H105, Y162, E167, E202, and H205) were replaced by alanine residues (E202A/H205A). The alkane degradation activity of mutant GK2771 (E202A/H205A) was compared with that of the non-mutated GK2771 at 37 °C (Fig. 5b). The C10–C19 alkane degradation activity of the mutant GK2771 (E202A/H205A) was slightly, but apparently, decreased. The differences between the remaining alkane amounts after treatment with GK2771 and GK2771 (E202A/H205A) were 13% (C10), 11% (C12), 13% (C14), 12% (C16), 21% (C18), and 20% (C19). This activity was not significantly lost by the E202A/H205A mutation, probably because metal ions were still partially bound to the mutant enzyme by the remaining six ligand amino acid residues. *Gk*R2loxI generally contains Fe and Mn at metal coordination sites 1 and 2, respectively. Mn and Fe occupy 90% and 10% of site 1. On the other hand, they occupy 70% and 30% of site 2, respectively, suggesting structural flexibility of site 2 (Kutin et al. 2019). Since E202 and H205 are amino acid ligands comprising the site 2, plasticity against these amino acid mutations may be possible.

*Gk*R2loxI produced in *E. coli* is purified as long-chain hydroxy fatty acids bound at the catalytic cleft (Griese et al. 2013). The structures of the long-chain hydroxy fatty acids bound to the enzyme were further carefully determined. 12-hydroxy-9-octadecenoic acid bound to the *Gk*R2loxI active site and no further transformation was observed. The positions of the hydroxyl residue, C_12_–OH, and the double bond, C_9_ = C_10_, were far from the oxygen-binding heterodinuclear Mn–Fe catalytic center in this octadecenoic acid. This suggests that octadecanoic acid is unlikely to be a substrate of *Gk*R2loxI. Moreover, *Mt*R2lox (*Mycobacterium tuberculosis* R2lox) and *Se*R2lox (*Saccharopolyspora erythraea* R2lox) were also produced in *E. coli* and tested for aldehyde deformylating oxygenase (ADO) activity. No such activity was detected for either of the enzymes. This indicates that fatty aldehydes are not R2lox substrates (Mak et al. 2020; Diamanti et al. 2022).

## Concluding remarks

*Geobacillus kaustophilus* HTA426 degrades C10–C24 alkanes, despite not having any alkane monooxygenase/hydroxylase gene homolog in the genome. The GK2771 protein, *Gk*R2loxI, was suggested to be the missing alkane monooxygenase/hydroxylase based on the structure of its gene cluster, which encodes a series of fatty alcohol-, aldehyde-, and fatty acyl-CoA-metabolizing enzymes. Previous information that medium-chain fatty acids are co-purified with *Gk*R2loxI also supports this possibility. In fact, GK2771-producing *E. coli* cell-free lysate degraded medium- to long-chain alkanes. GK2771 is a novel alkane monooxygenase/hydroxylase with a heterodinuclear Mn–Fe catalytic center. In addition to GK2771, *G. kaustophilus* HTA426 contains another R2lox protein *Gk*R2loxII, GKP26 in its plasmid, pHTA426. The GKP26 encoding gene is also accompanied by alcohol dehydrogenase (GKP30), aldehyde dehydrogenase (GKP31), and 4-chlorobenzoyl CoA ligase (GKP25)-encoding genes in the flanking region. The identity between GK2771 and GKP26 amino acid sequences is only 30%. Whether GKP26 is involved in aromatic hydrocarbon hydroxylation needs to be further examined. This study revealed for the first time that one of the functions of the R2loxI protein group is the aerobic hydroxylation of alkanes. A recombinant R2lox from the hyperthermophilic archaeum *Sulfolobus acidocaldarius*, *Sa*R2loxI, was also co-purified with fatty acids from *E. coli* cell lysates (Diamanti et al. 2022), suggesting that it also functions as an alkane monooxygenase.

## Data Availability

*G. kaustophilus* HTA426 complete genome sequence is available at ENA and Genbank/EMBL/DDBJ under accession numbers GCA_000009785.1 and BA000043.1 AP006520.1, respectively.
